# Supplementary material for: Analysis of PPARγ Signaling Activity in Psoriasis
Source: Int J Mol Sci. 2021 Aug 10;22(16):8603. doi: 10.3390/ijms22168603 (PMC8395241; doi:10.3390/ijms22168603)
Supplement: Supplementary file 1 [file ijms-22-08603-s001.zip › Supplemental materials_Analysis of PPARg signaling activity in psoriasis/Pathway models/Models images and html files/Interleukin-17 and interleukin-22 signaling in psoriasis/9331.html]

MAPK10


# Protein MAPK10

|  |  |
| --- | --- |
| URN | urn:agi-llid:5602 |
| Total Entities | 0 |
| Connectivity | 1180 |
| Name | MAPK10 |
| Description | mitogen-activated protein kinase 10 |
| Notes | The protein encoded by this gene is a member of the MAP kinase family. MAP kinases act as integration points for multiple biochemical signals, and thus are involved in a wide variety of cellular processes, such as proliferation, differentiation, transcription regulation and development. This kinase is specifically expressed in a subset of neurons in the nervous system, and is activated by threonine and tyrosine phosphorylation. Targeted deletion of this gene in mice suggests that it may have a role in stress-induced neuronal apoptosis. Alternatively spliced transcript variants encoding different isoforms have been described for this gene. A recent study provided evidence for translational readthrough in this gene, and expression of an additional C-terminally extended isoform via the use of an alternative in-frame translation termination codon. [provided by RefSeq, Dec 2017] |
| Primary Cell Localization | Cytoplasm |
| Class | Protein kinase |

---

|  |  |
| --- | --- |
| Pathway | Osteoarthritis |
|  | Skin Aging |
|  | Dopamine Receptors Signaling |
|  | Vascular Endothelial Cell Activation by Cytokines |
|  | Nociception Expression Targets Signaling |
|  | CNR1/2 -> IL1B/2/4/6/10 Production |
|  | DRD2 Expression Targets |
|  | Dendritic Cells Function in Atherosclerosis |
|  | Myocardial Infarction |
|  | Inflammatory Reaction in Acne Vulgaris |
|  | IL17 Signaling in Psoriasis |
|  | Dendritic Cells Function in Psoriasis |
|  | OipA Signaling in Helicobacter Pylori Infection |
|  | Osteoclast Activation in Multiple Myeloma |
|  | Hepatocellular Carcinoma |
|  | Growth Factor Signaling in Hepatocellular Carcinoma |
|  | Pancreatic Ductal Carcinoma |
|  | Growth Factor Signaling in Pancreatic Neoplasms |
|  | Pancreatic Neuroendocrine Tumors |
|  | TGFBR Signaling in Pancreatic Neoplasms |
|  | CFTR Facilitates Oxidative Stress in Airway Epithelium in CF |
|  | TNF and IL1B Induce Metalloproteinase Synthesis in Osteoarthritis |
|  | Skin Fibrosis |
|  | CCR5 Signaling in Macrophages |
|  | Glioma Invasion Signaling |
|  | WNT Signaling in Breast Cancer |
|  | ESR1/ERBB Positive Luminal Breast Cancer |
|  | ESR1 Signaling in Breast Cancer |
|  | Endometrial Cancer |
|  | Endometrioid Endometrial Cancer |
|  | Clear Cell Endometrial Cancer and Papillary Serous Endometrial Cancer |
|  | Angiogenesis in Endometriosis |
|  | Endometrioid Ovarian Carcinoma |
|  | Clear Cell Ovarian Carcinoma |
|  | High-Grade Serous Ovarian Carcinoma |
|  | Low-Grade Serous Ovarian Carcinoma |
|  | Ovarian Cancer |
|  | Mucinous Ovarian Carcinoma |
|  | Subepithelial Fibroblasts in Middle Ear Cholesteatoma |
|  | HPV E5 Regulates EDNRA and EGFR Signaling Pathway in Keratinocytes |
|  | Androgen Receptor/Akt Signaling |
|  | Steroids Induced Cataract |
|  | Cytokines Trigger Otitis Media |
|  | WNT Signaling in Cystic Kidney Disease |
|  | Apoptosis of Renal Tubule Epithelial Cell in Pyelonephritis |
|  | ANGPT2 -> AP-1/CREBBP/MYC Expression Targets |
|  | AREG -> AP-1 Expression Targets |
|  | Dopamine/Gi Expression Targets |
|  | BTC -> AP-1/ATF/CREB Expression Targets |
|  | AGTR1 -> ARRB1/ARRB2 Signaling |
|  | EGF -> AP-1/ATF Expression Targets |
|  | EGF -> CREB/CREBBP/ELK/SRF/MYC Expression Targets |
|  | EGF -> TP53 Expression Targets |
|  | EGFR/ERBB2 -> TP53 Signaling |
|  | EGFR -> AP-1/ATF2 Signaling |
|  | EGFR -> AP-1/CREB/ELK/SRF/MYC Signaling |
|  | EREG -> AP-1/ATF Expression Targets |
|  | Erythropoietin -> AP-1/MYC/CREB Expression Targets |
|  | F2 -> AP-1/CREB/ELK/SRF/SP1 Expression Targets |
|  | PLG -> AP-1/CREB/ELK/SRF/SP1 Expression Targets |
|  | FGF9 -> AP-1/CREB/MYC Expression Targets |
|  | FGF10 -> AP-1/CREB/CREBBP/MYC Expression Targets |
|  | FGF1 -> AP-1/CREB/ELK/SRF/MYC Expression Targets |
|  | FGF8 -> AP-1/CREB/MYC Expression Targets |
|  | FGF18 -> AP-1/CREB Expression Targets |
|  | FGF4 -> AP-1/MYC Expression Targets |
|  | FGF7 -> AP-1/CREB/CREBBP/MYC Expression Targets |
|  | FGF2 -> AP-1/CREB/CREBBP/ELK/SRF/MYC Expression Targets |
|  | FGFR -> AP-1/CREB/CREBBP/ELK/SRF/MYC Signaling |
|  | FrizzledR -> JUN/PAX2 Signaling |
|  | HBEGF -> AP-1/ATF Expression Targets |
|  | HBEGF -> TP53 Expression Targets |
|  | HBEGF -> CREB/MYC Expression Targets |
|  | HGF -> AP-1/CREB/ELK/SRF/MYC Expression Targets |
|  | HGFR -> AP-1/CREB/MYC Signaling |
|  | RAS/RAF/MAPK Signaling Activation in Cancer |
|  | RAS/RAF/MAPK Signaling Activation by Blocking of Tumor Suppressors |
|  | Apoptosis Block by Death Receptor Signaling in Cancer |
|  | Apoptosis Evasion in Cancer: Overview |
|  | Apoptosis Block by BCL2 Family Proteins in Cancer |
|  | VEGFA Dependent Angiogenesis in Cancer |
|  | VEGF Independent Angiogenesis in Cancer |
|  | FibronectinR -> AP-1/ELK/SRF/SREBF Signaling |
|  | CTGF -> AP-1/CREB/MYC Expression Targets |
|  | PAF/Gq -> AP-1/ATF1/CREB/ERK/SRF Expression Targets |
|  | Dronabinol/Anandamide Expression Targets |
|  | S1P Expression Targets |
|  | NRG1 -> CREB/CREBBP/ELK/SRF/MYC Expression Targets |
|  | NRG1 -> TP53 Expression Target |
|  | NRG1 -> AP-1/ATF Expression Targets |
|  | NGF -> AP-1/TP53/MYC Expression Targets |
|  | BDNF Expression Targets |
|  | NTF3 Expression Targets |
|  | NTF4 Expression Targets |
|  | PDGFB -> AP-1/CREB/MYC Expression Target |
|  | PDGFD -> AP-1 Expression Targets |
|  | PDGF -> AP-1/CREB/CREBBP/MYC Expression Targets |
|  | PDGFR -> AP-1/MYC Signaling |
|  | AVP/Gs -> CREB/ELK/SRF/AP-1/EGR Expression Targets |
|  | EDN1 Expression Targets |
|  | EDN3 Expression Targets |
|  | AVP/Gq -> CREB/ELK/SRF/AP-1/EGR Expression Targets |
|  | AngiopoietinR -> AP-1 Signaling |
|  | NTRK -> AP-1/CREB/ELK/SRF/MYC/SMAD3/TP53 Signaling |
|  | GAS6 -> AP-1/CREB Expression Targets |
|  | FIGF -> AP-1 Expression Target |
|  | KITLG -> AP-1/CREB/CREBBP/MYC Expression Targets |
|  | CSF1 -> AP-1/CREB/CREBBP/MYC Expression Targets |
|  | PGF -> AP-1/CREB/CREBBP/MYC Expression Targets |
|  | FLT3LG -> AP-1/CREB/CREBBP Expression Targets |
|  | TGFBR -> AP-1 Signaling |
|  | TNF-alpha/TNFRSF1A Signaling |
|  | JNK/MAPK Signaling |
|  | VEGF Signaling |
|  | EGFR Signaling |
|  | WNT Planar Cell Polarity (PCP) non-Canonical Signaling |
|  | TGFA -> TP53 Expression Targets |
|  | TGFA/AP-1/ATF Expression Targets |
|  | TGFA -> CREB/CREBBP/ELK-SRF/MYC Expression Targets |
|  | TGFB3-TGFBR2 Expression Targets |
|  | TGFB2-TGFBR2 Expression Targets |
|  | TGFB1-TGFBR2 Expression Targets |
|  | FASLG Expression Targets |
|  | TNFSF10 Expression Targets |
|  | LTA Expression Targets |
|  | TNFSF14 Expression Targets |
|  | TNF -> AP-1 Expression Targets |
|  | CD72 -> AP-1 Expression Targets |
|  | TNFSF13 Expression Targets |
|  | TNFSF13B Expression Targets |
|  | TNF -> TP53/ATF Expression Targets |
|  | EDA Expression Targets |
|  | EctodysplasinR -> AP-1 Signaling |
|  | TNFR -> AP-1/ATF/TP53 Signaling |
|  | NGFR -> AP-1/CEBPB/CREB/ELK/SRF/TP53 Signaling |
|  | TNFRSF1A -> AP-1/ATF/TP53 Signaling |
|  | Thrombopoietin -> AP-1/CREB/CREBBP/MYC Expression Targets |
|  | TLR4 -> AP-1 Expression Targets |
|  | TLR5 Expression Targets |
|  | TLR7 Expression Targets |
|  | TLR4 -> AP-1/EGR1/HIF1A Expression Targets |
|  | TLR1 -> 2/6 Expression Targets |
|  | TLR9 Expression Targets |
|  | TLR -> AP-1 Signaling |
|  | VEGFA -> AP-1/CREBBP/MYC Expression Targets |
|  | VEGFR -> AP-1/CREB/MYC Signaling |
|  | WNT1 Expression Targets |
|  | WNT3A Expression Targets |
|  | WNT4 Expression Targets |
|  | WNT5A Expression Targets |
|  | WNT7A Expression Targets |
|  | 1\_Inflammation in keratinocytes in the pathology of acne |
|  | 2\_Interleukin-17 and interleukin-22 signaling in psoriasis |
|  | 1\_TNF and IL1B provoke ECM degradation in osteoarthritis |
|  | 1\_Osteoclast-mediated bone resorption in postmenopausal women |
|  | 2\_Intestine epithelial cells drive immunological responses in ulcerative colitis |
|  | 2\_Neuron and Schwann cell death |
|  | mapk1 protein modificators |
|  | Interleukin-17 and interleukin-22 signaling in psoriasis |

---

|  |  |
| --- | --- |
| Group | Genes Associated with in Epileptic Encephalopathies |

---

|  |  |
| --- | --- |
| MedScan ID | 5602 |

---

|  |  |
| --- | --- |
| LocusLink ID | 5602 |
|  | 26414 |
|  | 25272 |

---

|  |  |
| --- | --- |
| Alias | JNK3 alpha protein kinase |
|  | c-Jun kinase 3 |
|  | p54b SAPK |
|  | stress activated protein kinase JNK3 |
|  | mitogen activated protein kinase 10 |
|  | OTTMUSP00000018410 |
|  | SAPK/Erk/kinase II |
|  | FLJ12099 |
|  | C230008H04 |
|  | OTTHUMP00000161181 |
|  | FLJ33785 |
|  | c-Jun N-terminal kinase 3 |
|  | protein kinase mitogen-activated 10 |
|  | SAPK1b |
|  | Serk2 |
|  | JNK3 beta1 protein kinase |
|  | C230008H04Rik |
|  | OTTHUMP00000161180 |
|  | JNK3 alpha2 protein kinase |
|  | JNK3 |
|  | JNK3A2 |
|  | SAPb |
|  | RIKEN cDNA C230008H04 gene |
|  | OTTMUSP00000018411 |
|  | p54-beta |
|  | c-Jun N-terminal kinase III |
|  | stress-activated protein kinase 1b |
|  | SAPKC |
|  | stress activated protein kinase beta |
|  | MAPK10 |
|  | MK10\_HUMAN |
|  | p54bSAPK |
|  | MAP kinase p49 3F12 |
|  | JNK3B2 |
|  | p49JNK3 |
|  | JNK3 beta2 protein kinase |
|  | p49SAPK |
|  | MK10 |
|  | p49MAPK |
|  | MGC50974 |
|  | OTTMUSP00000018409 |
|  | c-Jun kinase III |
|  | MAP kinase 10 |
|  | SAPK/Erk/kinase 2 |
|  | OTTMUSP00000018412 |
|  | OTTMUSP00000017557 |
|  | RP13-514E23.1 |
|  | JNK3A |
|  | OTTHUMP00000161182 |
|  | p493F12 |
|  | OTTHUMP00000161183 |
|  | JNK3B1 |
|  | kinase alpha-2 |
|  | SAPK(beta) |
|  | PRKM10 |
|  | stress-activated protein kinase JNK3 |
|  | mitogen-activated protein kinase 10 |
|  | MAPK 10 |
|  | SAPK-beta |

---

|  |  |
| --- | --- |
| EC Number | 2.7.11.24 |

---

|  |  |
| --- | --- |
| GO ID | 0005524 |
|  | 0004705 |
|  | 0004707 |
|  | 0004708 |
|  | 0038095 |
|  | 0007254 |
|  | 0007258 |
|  | 0000187 |
|  | 0071474 |
|  | 0071310 |
|  | 0035556 |
|  | 0000122 |
|  | 0098969 |
|  | 0006468 |
|  | 0051090 |
|  | 0042752 |
|  | 0010468 |
|  | 0009416 |
|  | 0009414 |
|  | 0048511 |
|  | 0007165 |
|  | 0099003 |
|  | 0005737 |
|  | 0005829 |
|  | 0098978 |
|  | 0005739 |
|  | 0043005 |
|  | 0005654 |
|  | 0005634 |
|  | 0043204 |
|  | 0005886 |
|  | 0016301 |
|  | 0000166 |
|  | 0004672 |
|  | 0004674 |
|  | 0016740 |
|  | 0045475 |
|  | 0016310 |
|  | 0016020 |
|  | 0014069 |
|  | 0000165 |
|  | 0097481 |
|  | 0005622 |
|  | 0002755 |
|  | 0002756 |
|  | 0035666 |
|  | 0045087 |
|  | 0051403 |
|  | 0034166 |
|  | 0034134 |
|  | 0034138 |
|  | 0034142 |
|  | 0034146 |
|  | 0034162 |
|  | 0038123 |
|  | 0038124 |
|  | 0002224 |
|  | 0016772 |

---

|  |  |
| --- | --- |
| KEGG ID | hsa:5602 |
|  | mmu:26414 |
|  | rno:25272 |

---

|  |  |
| --- | --- |
| Organism | Homo sapiens {Organism urn:agi-taxid:9606} |
|  | Mus musculus {Organism urn:agi-taxid:10090} |
|  | Rattus norvegicus {Organism urn:agi-taxid:10116} |
|  | Homo sapiens |
|  | Mus musculus |
|  | Rattus norvegicus |

---

|  |  |
| --- | --- |
| Mouse chromosome position | 5 |

---

|  |  |
| --- | --- |
| OMIM ID | 602897 |

---

|  |  |
| --- | --- |
| Rat chromosome position | 14p22 |

---

|  |  |
| --- | --- |
| Hugo ID | 6872 |
|  | HGNC:6872 |

---

|  |  |
| --- | --- |
| Human chromosome position | 4q21.3 |
|  | 4q22.1-q23 |

---

|  |  |
| --- | --- |
| Swiss-Prot Accession | A0A286YF97 |
|  | P53779 |
|  | Q499Y8 |
|  | A8MWW6 |
|  | A0A286YFD7 |
|  | I6L9F3 |
|  | A0A1P0B7D2 |
|  | D6RJF9 |
|  | A0A286YEQ7 |
|  | A0A286YFN2 |
|  | A0A286YES0 |
|  | A0A286YF02 |
|  | D6RCB1 |
|  | P53779.2 |
|  | Q78GB8 |
|  | Q8C9D4 |
|  | Q80W80 |
|  | Q61831 |
|  | Q3TQZ7 |
|  | Q80W81 |
|  | Q80W82 |
|  | Q61831.2 |
|  | A0A0U1RVI2 |
|  | B0VXR6 |
|  | P49187 |
|  | P49187.3 |
|  | A6NFS3 |
|  | A6NG28 |
|  | B3KQ94 |
|  | Q15707 |
|  | Q49AP1 |
|  | Q9R0U6 |
|  | D3ZQ33 |
|  | P49187.2 |

---

|  |  |
| --- | --- |
| PIR ID | S71104 |
|  | S43969 |

---

|  |  |
| --- | --- |
| GenBank ID | NC\_000004 |
|  | XR\_001741289 |
|  | NM\_138982 |
|  | NP\_620448 |
|  | NM\_001318069 |
|  | NP\_001304998 |
|  | NM\_001351624 |
|  | NP\_001338553 |
|  | NM\_138980 |
|  | NP\_620446 |
|  | NM\_002753 |
|  | NP\_002744 |
|  | NM\_001351625 |
|  | NP\_001338554 |
|  | NM\_001363657 |
|  | NP\_001350586 |
|  | NM\_001318068 |
|  | NP\_001304997 |
|  | NM\_001318067 |
|  | NP\_001304996 |
|  | XM\_024454141 |
|  | XP\_024309909 |
|  | XM\_017008428 |
|  | XP\_016863917 |
|  | XM\_011532120 |
|  | XP\_011530422 |
|  | XM\_024454142 |
|  | XP\_024309910 |
|  | XM\_024454145 |
|  | XP\_024309913 |
|  | XM\_011532117 |
|  | XP\_011530419 |
|  | XM\_024454144 |
|  | XP\_024309912 |
|  | XM\_024454140 |
|  | XP\_024309908 |
|  | XM\_024454148 |
|  | XP\_024309916 |
|  | XM\_024454139 |
|  | XP\_024309907 |
|  | XM\_017008448 |
|  | XP\_016863937 |
|  | XM\_005263131 |
|  | XP\_005263188 |
|  | XM\_006714269 |
|  | XP\_006714332 |
|  | XM\_005263135 |
|  | XP\_005263192 |
|  | XM\_017008434 |
|  | XP\_016863923 |
|  | XM\_017008450 |
|  | XP\_016863939 |
|  | XM\_017008433 |
|  | XP\_016863922 |
|  | XM\_017008441 |
|  | XP\_016863930 |
|  | XM\_017008427 |
|  | XP\_016863916 |
|  | XM\_017008445 |
|  | XP\_016863934 |
|  | XM\_017008451 |
|  | XP\_016863940 |
|  | XM\_017008449 |
|  | XP\_016863938 |
|  | XM\_017008447 |
|  | XP\_016863936 |
|  | XM\_017008452 |
|  | XP\_016863941 |
|  | XM\_017008420 |
|  | XP\_016863909 |
|  | XM\_006714268 |
|  | XP\_006714331 |
|  | XM\_017008435 |
|  | XP\_016863924 |
|  | XM\_017008429 |
|  | XP\_016863918 |
|  | XM\_005263129 |
|  | XP\_005263186 |
|  | XM\_017008430 |
|  | XP\_016863919 |
|  | XM\_017008423 |
|  | XP\_016863912 |
|  | XM\_017008436 |
|  | XP\_016863925 |
|  | XM\_011532118 |
|  | XP\_011530420 |
|  | XM\_024454146 |
|  | XP\_024309914 |
|  | XM\_011532121 |
|  | XP\_011530423 |
|  | XM\_017008437 |
|  | XP\_016863926 |
|  | XM\_005263130 |
|  | XP\_005263187 |
|  | XM\_017008432 |
|  | XP\_016863921 |
|  | XM\_024454143 |
|  | XP\_024309911 |
|  | XM\_024454147 |
|  | XP\_024309915 |
|  | XM\_017008422 |
|  | XP\_016863911 |
|  | XM\_017008454 |
|  | XP\_016863943 |
|  | XM\_024454149 |
|  | XP\_024309917 |
|  | XM\_017008453 |
|  | XP\_016863942 |
|  | NG\_013325 |
|  | AC096953 |
|  | AC104059 |
|  | AC104827 |
|  | AC108054 |
|  | AC110076 |
|  | AX027293 |
|  | CAC09125 |
|  | AX027295 |
|  | CAC09126 |
|  | CH471057 |
|  | EAX05962 |
|  | EAX05963 |
|  | CS693282 |
|  | CAP12256 |
|  | CS693286 |
|  | CAP12257 |
|  | CS693290 |
|  | CAP12259 |
|  | KT585173 |
|  | AF052141 |
|  | AK022161 |
|  | AK057723 |
|  | BAG51956 |
|  | AK091104 |
|  | BAG52284 |
|  | AK124791 |
|  | BAG54096 |
|  | AK225974 |
|  | AK294125 |
|  | BAH11677 |
|  | AK307129 |
|  | BC022492 |
|  | BC035057 |
|  | AAH35057 |
|  | BC051731 |
|  | AAH51731 |
|  | BC065516 |
|  | AAH65516 |
|  | BI549234 |
|  | CB069160 |
|  | DA192997 |
|  | U07620 |
|  | AAC50101 |
|  | U34819 |
|  | AAC50604 |
|  | U34820 |
|  | AAC50605 |
|  | P53779 |
|  | NC\_000071 |
|  | NM\_001081567 |
|  | NP\_001075036 |
|  | NM\_001318102 |
|  | NP\_001305031 |
|  | NM\_009158 |
|  | NP\_033184 |
|  | NM\_001310683 |
|  | NP\_001297612 |
|  | NM\_001310685 |
|  | NP\_001297614 |
|  | NM\_001318131 |
|  | NP\_001305060 |
|  | NM\_001310686 |
|  | NP\_001297615 |
|  | XM\_017320890 |
|  | XP\_017176379 |
|  | XM\_006534950 |
|  | XP\_006535013 |
|  | XM\_017320886 |
|  | XP\_017176375 |
|  | XM\_017320891 |
|  | XP\_017176380 |
|  | XM\_017320889 |
|  | XP\_017176378 |
|  | XM\_017320888 |
|  | XP\_017176377 |
|  | XM\_011249450 |
|  | XP\_011247752 |
|  | AC123664 |
|  | AC125252 |
|  | AC137121 |
|  | AC160244 |
|  | CH466529 |
|  | EDL20250 |
|  | EDL20251 |
|  | EDL20252 |
|  | AB005665 |
|  | BAA85877 |
|  | AB096076 |
|  | BAC76450 |
|  | AB096077 |
|  | BAC76451 |
|  | AB096078 |
|  | BAC76452 |
|  | AB096079 |
|  | BAC76453 |
|  | AK036275 |
|  | AK042368 |
|  | BAC31240 |
|  | AK046409 |
|  | BAC32713 |
|  | AK048693 |
|  | AK076990 |
|  | AK082242 |
|  | AK082729 |
|  | AK136881 |
|  | AK163206 |
|  | BAE37234 |
|  | AK163385 |
|  | BC046625 |
|  | AAH46625 |
|  | CA751768 |
|  | L35236 |
|  | AAB37741 |
|  | Q61831 |
|  | NC\_005113 |
|  | XM\_017599079 |
|  | XP\_017454568 |
|  | XM\_017599080 |
|  | XP\_017454569 |
|  | XM\_017599082 |
|  | XP\_017454571 |
|  | XM\_017599084 |
|  | XP\_017454573 |
|  | XM\_017599083 |
|  | XP\_017454572 |
|  | NM\_012806 |
|  | NP\_036938 |
|  | NM\_001270556 |
|  | NP\_001257485 |
|  | NM\_001318190 |
|  | NP\_001305119 |
|  | XM\_017599081 |
|  | XP\_017454570 |
|  | AC\_000082 |
|  | AABR07014301 |
|  | AABR07072369 |
|  | AAHX01077960 |
|  | AAHX01077961 |
|  | AAHX01077962 |
|  | AAHX01077963 |
|  | AAHX01077964 |
|  | AAHX01077965 |
|  | AAHX01077966 |
|  | AAHX01077967 |
|  | AAHX01077968 |
|  | AAHX01077969 |
|  | AAHX01077970 |
|  | AAHX01077971 |
|  | AAHX01077972 |
|  | AAHX01077973 |
|  | AAHX01077974 |
|  | AAHX01077975 |
|  | AC141145 |
|  | CH474022 |
|  | EDL99526 |
|  | EDL99527 |
|  | CS693293 |
|  | CAP12261 |
|  | DQ377224 |
|  | ABD24063 |
|  | FQ083521 |
|  | FQ084081 |
|  | FQ211777 |
|  | L27128 |
|  | AAA42110 |
|  | P49187 |
|  | XM\_011532119 |
|  | XP\_011530421 |
|  | XM\_011532122 |
|  | XP\_011530424 |
|  | XM\_005263136 |
|  | XP\_005263193 |
|  | NM\_138981 |
|  | NP\_620447 |
|  | XM\_011532123 |
|  | XP\_011530425 |
|  | XM\_006714272 |
|  | XP\_006714335 |
|  | XM\_006714271 |
|  | XP\_006714334 |
|  | NC\_018915 |
|  | AMYH02009652 |
|  | AMYH02009653 |
|  | AMYH02009654 |
|  | AMYH02009655 |
|  | HG497056 |
|  | XM\_006534946 |
|  | XP\_006535009 |
|  | XM\_006534951 |
|  | XP\_006535014 |
|  | XM\_006534949 |
|  | XP\_006535012 |
|  | XM\_011249449 |
|  | XP\_011247751 |
|  | AC\_000027 |
|  | AAHY01047444 |
|  | AAHY01047445 |
|  | AAHY01047446 |
|  | AAHY01047447 |
|  | AAHY01047448 |
|  | AAHY01047449 |
|  | AAHY01047450 |
|  | AAHY01047451 |
|  | AAHY01047452 |
|  | AAHY01047453 |
|  | AAHY01047454 |
|  | BC026697 |
|  | XM\_008769992 |
|  | XP\_008768214 |
|  | XM\_006714267 |
|  | XP\_006714330 |
|  | XM\_006714270 |
|  | XP\_006714333 |
|  | XM\_005263138 |
|  | XP\_005263195 |
|  | AC\_000136 |
|  | ABBA01063498 |
|  | ABBA01063499 |
|  | ABBA01063500 |
|  | XM\_006534944 |
|  | XP\_006535007 |
|  | XM\_006534943 |
|  | XP\_006535006 |
|  | XM\_006534947 |
|  | XP\_006535010 |
|  | XM\_006534945 |
|  | XP\_006535008 |
|  | XM\_006534948 |
|  | XP\_006535011 |
|  | XM\_006534952 |
|  | XP\_006535015 |

---

|  |  |
| --- | --- |
| Swiss-Prot ID | MK10\_HUMAN |
|  | MK10\_MOUSE |
|  | MK10\_RAT |

---

|  |  |
| --- | --- |
| Cell Localization | Cytoplasm |
|  | Membrane |
|  | Nucleus |
|  | Mitochondrion |

---

|  |  |
| --- | --- |
| Ensembl ID | ENSG00000109339 |
|  | ENSP00000493344.1 |
|  | ENST00000641718.1 |
|  | ENSP00000414469.2 |
|  | ENST00000449047.6 |
|  | ENSP00000493040.1 |
|  | ENST00000642015.1 |
|  | ENSP00000493237.1 |
|  | ENST00000641170.1 |
|  | ENSP00000491866.1 |
|  | ENST00000638225.1 |
|  | ENSP00000493435.1 |
|  | ENST00000641462.2 |
|  | ENSP00000493275.1 |
|  | ENST00000641051.1 |
|  | ENSP00000493177.1 |
|  | ENST00000641737.1 |
|  | ENSP00000493363.1 |
|  | ENST00000641157.1 |
|  | ENSP00000493270.1 |
|  | ENST00000641050.1 |
|  | ENSP00000493013.1 |
|  | ENST00000641952.1 |
|  | ENSP00000492913.1 |
|  | ENST00000641553.1 |
|  | ENSP00000378598.5 |
|  | ENST00000395169.9 |
|  | ENSP00000378586.4 |
|  | ENST00000395157.9 |
|  | ENSP00000352157.4 |
|  | ENST00000359221.8 |
|  | ENSP00000492898.1 |
|  | ENST00000641384.1 |
|  | ENSP00000493447.1 |
|  | ENST00000642023.1 |
|  | ENSP00000493350.1 |
|  | ENST00000641166.1 |
|  | ENSP00000492920.1 |
|  | ENST00000641607.1 |
|  | ENSP00000493455.1 |
|  | ENST00000641047.1 |
|  | ENSP00000492912.1 |
|  | ENST00000641881.1 |
|  | ENSP00000493045.1 |
|  | ENST00000641983.1 |
|  | ENSP00000493049.1 |
|  | ENST00000641803.1 |
|  | ENSP00000425654.2 |
|  | ENST00000512689.6 |
|  | ENSP00000491122.1 |
|  | ENST00000640858.1 |
|  | ENSP00000493072.1 |
|  | ENST00000641066.1 |
|  | ENSP00000492231.1 |
|  | ENST00000640970.1 |
|  | ENSP00000378595.1 |
|  | ENST00000395166.6 |
|  | ENSP00000493206.1 |
|  | ENST00000641989.1 |
|  | ENSP00000492292.1 |
|  | ENST00000638313.1 |
|  | ENSP00000492942.1 |
|  | ENST00000642038.1 |
|  | ENSP00000491089.1 |
|  | ENST00000639242.1 |
|  | ENSP00000493309.1 |
|  | ENST00000641767.1 |
|  | ENSP00000493396.1 |
|  | ENST00000641862.1 |
|  | ENSP00000422277.2 |
|  | ENST00000511167.6 |
|  | ENSMUSG00000046709 |
|  | ENSMUSP00000108468.2 |
|  | ENSMUST00000112847.8 |
|  | ENSMUSP00000084065.3 |
|  | ENSMUST00000086854.9 |
|  | ENSMUSP00000127193.1 |
|  | ENSMUST00000170792.7 |
|  | ENSRNOG00000002079 |
|  | ENSRNOP00000060904.4 |
|  | ENSRNOT00000065965.4 |
|  | ENSRNOP00000075846.1 |
|  | ENSRNOT00000092395.1 |
|  | ENSP00000355297 |
|  | ENST00000361569 |
|  | ENSP00000378598 |
|  | ENST00000395169 |
|  | ENSP00000352157 |
|  | ENST00000359221 |
|  | ENSP00000378590 |
|  | ENST00000395161 |
|  | ENSP00000378586 |
|  | ENST00000395157 |
|  | ENSMUSP00000108468 |
|  | ENSMUST00000112847 |
|  | ENSMUSP00000127193 |
|  | ENSMUST00000170792 |
|  | ENSMUSP00000084065 |
|  | ENSMUST00000086854 |
|  | ENSRNOP00000060904 |
|  | ENSRNOT00000065965 |
|  | ENSRNOP00000075846 |
|  | ENSRNOT00000092395 |

---

|  |  |
| --- | --- |
| MGI ID | MGI:1346863 |

---

|  |  |
| --- | --- |
| RGD ID | 3663 |

---

|  |  |
| --- | --- |
| Unigene ID | Hs.13438 |
|  | Hs.125503 |
|  | Mm.39253 |
|  | Mm.472459 |
|  | Rn.9911 |

---

|  |  |
| --- | --- |
| Homologene ID | 56439 |

---

|  |  |
| --- | --- |
| Shape | Sickle-vertex |

---
